# Supplementary material for: Pairtools: from sequencing data to chromosome contacts
Source: bioRxiv. 2023 Feb 15:2023.02.13.528389. Preprint. [Version 1] doi: 10.1101/2023.02.13.528389 (PMC9949071; doi:10.1101/2023.02.13.528389)
Supplement: Supplement 1 [file NIHPP2023.02.13.528389v1-supplement-1.pdf]

bioRxiv preprint doi: https://doi.org/10.1101/2023.02.13.528389; this version posted February 15, 2023. The copyright holder for this preprint (which was not certified by peer review) is the author/funder, who has granted bioRxiv a license to display the preprint in perpetuity. It is made available under aCC-BY 4.0 International license.

Supplementary Figures

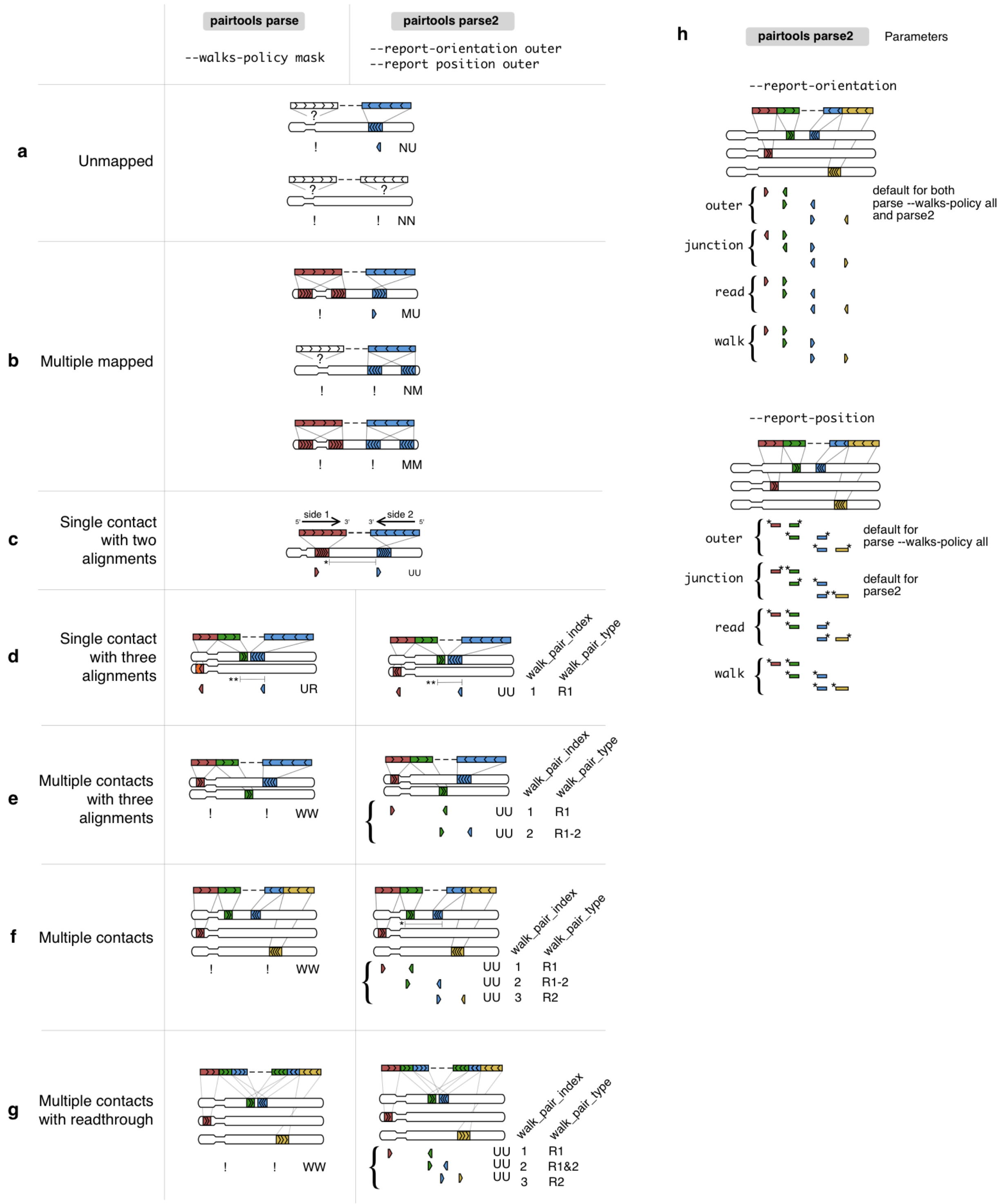

Supplementary Figure 1. Parameters of pairs parsing.

a-g. Different types of paired-end reads processed by *parse* and *parse2*. Notation is the same as in Figure 1b.

a. Unmapped reads. Either one (top) or both (bottom) sides of the read do not contain segments aligned to the reference genome.

b. Multiple mapped reads. Either one (top, center) or both (bottom) sides of the read contain a segment that is mapped to multiple locations in the genome.

c. Single contact with two alignments. Each side of the read contains a uniquely mapped alignment (red and blue). Alignments should either map to different chromosomes or at a distance larger than the expected molecule size (marked by *). The expected molecule size is a ***pairtools parse*** parameter and typically is around 500-800 bp for Hi-C.

d. Single contact with three alignments. One side of the reads contains two segments that are mapped to different unique locations in the reference genome (red and green), and the 3' segment of the read (green) is mapped to the location close to the alignment of

bioRxiv preprint doi: https://doi.org/10.1101/2023.02.13.528389; this version posted February 15, 2023. The copyright holder for this preprint (which was not certified by peer review) is the author/funder, who has granted bioRxiv a license to display the preprint in perpetuity. It is made available under aCC-BY 4.0 International license.

the second read side (blue). The distance is closer than molecule size (marked by **), thus green and blue segments are parts of the same continuous DNA fragment in the 3C+ chimeric molecule. Thus, this case is a single contact with three alignments. Both *parse* and *parse2* recognize it and report a single pair. *Parse* will mark this pair as "rescued".

e. Multiple contacts with three alignments. One side of the reads contains two segments that are mapped to different unique locations in the reference genome (red and green), but the 3' segment of the read (green) is mapped not very close to the alignment of the second read side (blue, mapped to another chromosome). Thus, this 3C+ chimeric molecule captures multiple contacts (two of them observed) that have three alignments. *Parse* will report such cases as unrecognized walks (W), and *parse2* will report both contacts (with different walk pair types).

f. Multiple contacts. Both sides of the read contain two segments mapped to different unique locations. *Parse* will report an unrecognized walk (W), and *parse2* will report three contacts (with different walk pair types). Note that the 3' segments on both sides are mapped to different locations in the reference genome that are further than the molecule size in the genome (marked with *). Also note that the original 3C+ DNA molecule, in this case, is longer than two sequencing lengths, resulting in an unsequenced region (marked with the dashed line between green and blue alignment).

g. Multiple contacts with readthrough (internal duplicates). Both sides of the read contain three segments. However, 3'-ends of the reads are reverse complements of each other, resulting in green and blue pairs being duplicated on both sides 1 and 2. In this case, the original 3C+ DNA molecule was shorter than two sequencing lengths, resulting in read-through. *Parse* will report an unrecognized walk (W), and *parse2* will detect the readthrough, perform internal deduplication and report three contacts (with different walk pair types).

h. Modes of reporting contacts in *parse2*. The alignment group under each bracket represents all the contacts reported for this read.

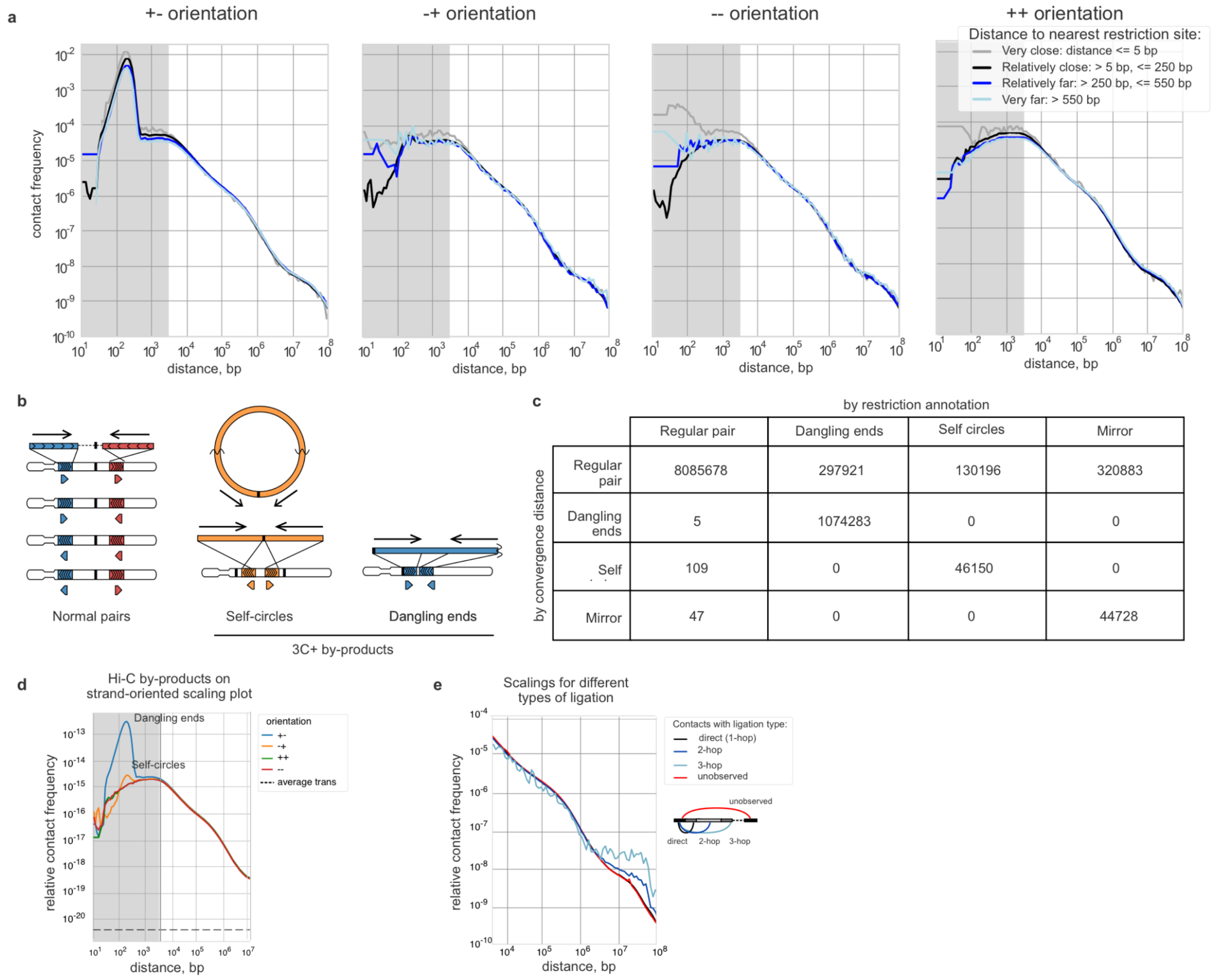

	Regular pair	Dangling ends	Self circles	Mirror
Regular pair	8085678	297921	130196	320883
Dangling ends	5	1074283	0	0
Self	109	0	46150	0
Mirror	47	0	0	44728

Supplementary Figure 2. Pairtools scaling and quality control of 3C+ data.

bioRxiv preprint doi: https://doi.org/10.1101/2023.02.13.528389; this version posted February 15, 2023. The copyright holder for this preprint (which was not certified by peer review) is the author/funder, who has granted bioRxiv a license to display the preprint in perpetuity. It is made available under aCC-BY 4.0 International license.

a. Orientation-dependent scalings for pairs grouped by distance to the nearest restriction site (DpnII Hi-C from [1]). Scalings are very close at genomic separations beyond the orientation convergence distance.
b. Generation of normal pairs and by-products in 3C+ protocol. Normal pairs originate from distinct restriction fragments separated by at least one restriction site (in black). Pairs in self-circles and dangling ends are located on the same restriction site, either in divergent (self-circles) or convergent (dangling ends) orientation.
c. Counts of pairs are categorized into four groups: regular pairs, dangling ends, self circles, and mirror pairs [47] for a test sample of 11 million pairs, by restriction enzyme annotation (columns) and convergence distance (rows). For restriction enzyme annotation, we considered dangling ends to be mapped to the same restriction fragment in the convergent orientation, self circles in the divergent orientation, and mirror pairs in the same orientation. For convergence distance annotation, we conservatively considered all the pairs below convergence distance as potential by-products and assigned them to each category by their orientation as for the restriction enzyme annotation. Both methods produce highly congruent filtration, as seen by the relatively smaller number of off-diagonal pairs.
d. Scaling with prominent peak of self-circles and dangling ends. A short-range peak in pairs mapped to opposing strands facing away from each other (divergent) is a sign of self-circled DNA, while a short-range peak in pairs mapped to opposing strands facing each other (convergent) pairs is a sign of dangling ends.
e. Scalings for direct, indirect (2- and 3-hops), and unobserved contacts. Note that multi-hop contacts have a flatter scaling, potentially indicating more ligations in the solution. [16] [17]
